# Levels of serum estradiol and lifestyle factors related with bone mineral density in premenopausal Mexican women: a cross-sectional analysis

**DOI:** 10.1186/s12891-016-1273-7

**Published:** 2016-10-19

**Authors:** Gerardo Huitrón-Bravo, Edgar Denova-Gutiérrez, Juan O. Talavera, Carlos Moran-Villota, Juan Tamayo, Arianna Omaña-Covarrubias, Jorge Salmerón

**Affiliations:** 1 Facultad de Medicina, Universidad Autónoma del Estado de México, Toluca, Estado de México Mexico; 2 Unidad de Investigación en Epidemiología Clínica, Hospital Infantil de México “Federico Gómez”, Calle Dr. Márquez No.162, Del. Cuahtemoc, Col. Doctores, C.P., 06720 Ciudad de México, Mexico; 3 Unidad de Investigación Médica en Epidemiología Clínica, Hospital de Especialidades Centro Médico Nacional Siglo XXI, Instituto Mexicano del Seguro Social, Ciudad de México, Mexico; 4 Unidad de Investigación Médica en Medicina Reproductiva, Instituto Mexicano del Seguro Social, Ciudad de México, Mexico; 5 Comité Mexicano para la prevención de la Osteoporosis, Ciudad de México, Mexico; 6 Área Académica de Nutrición, Universidad Autónoma del Estado de Hidalgo, Pachuca, Hidalgo Mexico; 7 Unidad de Investigación Epidemiológica y en Servicios de Salud, Instituto Mexicano del Seguro Social, Morelos, Mexico; 8 Centro de Investigación en Salud Poblacional, Instituto Nacional de Salud Pública, Cuernavaca, Morelos Mexico

**Keywords:** Serum estradiol, Bone mineral density, Premenopausal, Mexican women

## Abstract

**Background:**

Many factors, such as heredity, ethnicity, nutrition and other lifestyle factors, have been related to bone mineral density in postmenopausal women. Additionally, bone mass has been significantly associated with decreased estrogen levels. However, fewstudies have been conducted on premenopausal women. The present study was designed to estimate the relationship between low bone mineral density and levels of serum estradiol and lifestyle factors in premenopausal Mexican women.

**Methods:**

A cross-sectional study was conducted in 270 women between 40 and 48 years of age who participate in the Health Workers Cohort Study. Information on socio-demographic and lifestyle factors were obtained through a self-administered questionnaire. Body mass index and serum estradiol were measured with standard procedures; bone mineral density was assessed using dual-energy X-ray absorptiometry. Multiple linear and logistic regression models were computed to evaluate the relationship between low bone mineral density and levels of serum estradiol and lifestyle factors.

**Results:**

In linear regression analysis levels of estradiol, body mass index, physical activity, and vitamin D intake were positively related to bone mineral density. Age, cigarette smoking and caffeine were inversely associated with BMD. Finally, the odds of low bone mineral density increase significantly when the premenopausal women had low levels of serum estradiol (OR = 4.93, 95 % CI: 2.14, 11.37).

**Conclusion:**

These data support that low serum estradiol, advancing age, lower physical activity, lower vitamin D intake, cigarette smoking, and higher amount of caffeine intake are linked to low bone mineral density in premenopausal Mexican women.

## Background

Osteoporosis is a skeletal condition represented by low bone mineral density and the depreciation of the microarchitecture of the bone tissue [1]. While this disorder is common among the elderly, it is also a problem among younger people. For example, in premenopausal women, about 15 % have bone mineral density levels below −1 standard deviation, and about 0.6 % are more than −2.5 standard deviations below young-adult mean bone mineral density [2]. Globally, osteoporosis is one of the most important public health problems, predisposing people to fractures [3–6], which are associated with increased morbidity and mortality [3, 4], reduced quality of life [5] and pose high health-care costs [6].

Bone mineral density (BMD) in women has been related to many factors such as age at menarche, number of pregnancies, breastfeeding, and contraceptive use; as well as physical activity, tobacco use and intakes of calcium, caffeine, dietary fiber, dietary vitamin D and alcohol [7–11].

Perimenopause is an intermediate phase in a woman’s life when physiological changes occur that begin the transition to menopause, such as irregular menstrual periods [12, 13]. In Mexican women, it has been observed that after the age of 40 years bone mass decreases gradually [14], which could be associated with lowered estrogen production that happens several years prior to the start of the menopause. On the other hand, estrogens play an important role in bone growth and homeostasis [15]. Furthermore, in women, levels of sex steroids are a significant component responsible for bone turnover and bone density [16], especially when estrogen production reduces and levels fall, accelerated bone loss ensues.

Therefore, our study was designed to evaluate the relationship between bone mineral density and levels of serum estradiol and lifestyle factors in premenopausal Mexican women.

## Methods

### Study population

The present study included a sample of adult females (40–48 years old) participating in the Health Workers Cohort Study (HWCS). In short, the HWCS include healthy members from health and academic institutions located in two cities of central Mexico: Instituto Mexicano del Seguro Social (IMSS) and the Instituto Nacional de Salud Pública (INSP) and Universidad Autónoma del Estado de México (UAEMex). In general, the main objective of the HWCS is to evaluate the associations between different lifestyle factors and health outcomes in the Mexican population. The specifics about methods and participants’ characteristics have been described previously [17–19].

From March 2004 to April 2006, a total of 9,467 subjects were requested to participate in the HWCS, of these, 8,307 adults were registered in the cohort study. Of those, the female population of the HWCS consists of 6,240 women ranging from 18 to 90 years. Due to the nature of the study, only women working at the UAEMex were included, individuals with the following characteristics were excluded from the final analysis: females who work at IMSS, or at INSP (*n* = 4392); women under 40 years and over 48 years (*n* = 1439); subjects reporting current or past occurrence of any medical conditions recognized to influence bone metabolism (*n* = 26); and women who had been taking any medication that affects bone density, for example, glucocorticoids (*n* = 84). We also excluded subjects who did not have complete information (*n* = 29). In total, 270 females were included in the final analysis. All of them continued with regular menstrual cycles.

All women participating in the study gave written informed consent to the procedures, which were reviewed and accepted by the ethics committee of the Medical Science Research Center (No. 1233008X0236). Finally, the present study was conducted following the recommendations of the Declaration of Helsinki.

### Measures

Females included in the present study gave information on socio-demographic characteristics, reproductive factors (such as: number of pregnancies, breastfeeding, and contraceptive use) and lifestyle information on dietary intake, use of multivitamin supplements, physical activity, tobacco use and family history of osteoporosis.

### Physical activity measurement

The International Physical Activity Questionnaire (IPAQ) [20], which has been previously adjusted for the Mexican population, was used to assess habitual physical activity. Subjects answered questions concerning the period of time they assigned to physical activity throughout leisure time, at work and doing housework. Physical activity was measured in metabolic equivalents (METS) and was estimated according to the time and intensity performed in different activities like walking, dancing, swimming, and doing other exercises for 1 week, in the year previous to the study. We have also evaluated participants’ sedentary activities, defined as time devoted to activity with low METS. Sedentary activities included reading, writing, working at the computer, watching TV, going to the movies, activity planning and playing table games.

### Dietary intake measurement

A food frequency questionnaire (FFQ) previously validated in Mexican adult population [21] was employed to evaluate dietary intake. The FFQ included information on the average frequency of intake of 116 food items during the previous year. Dietary vitamin D (UI/d) as well as dietary calcium (mg/d) consumption were estimated using the FFQ above mentioned. Multivitamin supplement use (including calcium) was determined accordant to names and daily doses reported by participants. Calcium and vitamin D intake were determined by the sum of dietary calcium consumption plus vitamin D and calcium from supplement consumption. Finally, both, the consumption of dietary vitamin D and calcium were estimated by multiplying the frequency of consumption of each food by the vitamin D and calcium content of the specified portion size. Additionally, Alcohol and caffeine consumption were assessed as drinks/day, from which g/day and mg/day, respectively, were calculated. By means of a food contents database we computed the specific nutrient intakes [22].

Using the standard deviation method suggested by Rosner [23], the outlier values in energy intake (values below 600 kcal/day and above 7000 kcal/day) were eliminated from the final analysis. All nutrient intakes were adjusted by the residual method.

### Tobacco use and familial history of osteoporosis

Tobacco use was assessed by means of a self-reported questionnaire, constructed on previous and current cigarette smoking. Tobacco use was categorized as “yes” for current smoking and past smoking, or “no” for never smoking. Additionally, a female was defined as a smoker, if she informed consuming at least 100 cigarettes in her lifetime [17, 18].

If a woman responded yes to the question “Has anyone in your family ever had osteoporosis?” She was classified as a positive to familial history of osteoporosis [18].

### Reproductive factors measurement

The reproductive factors like: age at menarche, duration of menstrual cycles, use of contraceptive, number of gestations, and duration of breastfeed were assessed. Age at menarche was defined as the age of the first menstrual bleeding. Duration of menstrual cycles was described as the time in days from the first day of menstruation to the initiation of the following period. In our study population all women continued with their menstrual cycle. The use of oral contraceptive was expressed as the accrued time since women took oral contraceptives. We obtained information about the number of gestations (including only full-term pregnancies), which were dichotomized as < 2, and ≥ 2 gestations, to the final analysis. Lastly, the time of breastfeed was assessed by the calculation of total months that a woman gave breastfeeding to her different children, for the present analysis, two groups were established: < 6 months and ≥ 6 months [24].

### Anthropometric measurements

Body weight (kg) was evaluated with an electronic TANITA scale (model BC-533, Tokyo, Japan), formerly calibrated, with females dressing minimal clothes. Body height (m) was assessed by means of a stadiometer. Using a measuring tape, waist circumference was calculated at the high point of the iliac crest at the end of normal expiration; to the nearest 0.1 cm. Body mass index was calculated as a ratio of weight (kg) to height in meters squared (BMI, kg/m^2^). For the present study, BMI was utilized to categorize women as follows: normal BMI (≥18.5 to <25.0 kg/m^2^); overweight (≥25.0 to <30.0 kg/m^2^) and obese (≥30.0 kg/m^2^) [25].

### Levels of serum estradiol

A blood sample was drawn between 7 and 9 am into evacuated tubes after subjects fasted overnight (at least 12 h). Levels of serum estradiol (pg/mL) were assessed through competitive immunoassay (Immulite estradiol, Diagnostic Products Corp., Los Angeles, CA) [26]. Immulite is a computerized, random-access immunoassay analyzer with a solid-phase and a competitive chemiluminescent enzyme. The solid stage (bead) is covered with rabbit anti-estradiol polyclonal antibody. The women’s serum test and alkaline phosphatase-conjugated are concurrently insert into the test unit. During 60-min incubation at 37 °C with intermittent shaking, estradiol in the patient sample competes with the enzyme-labeled estradiol for a partial quantity of antibody binding sites on the bead. Chemiluminescent substrate, a phosphate ester of adamantyl dioxetane, is inserted and the test unit is incubated for 10 min. The substrate is hydrolyzed by alkaline phosphatase to an unstable anion. The disintegration of the anion yields a sustained release of light. The bound complex, corresponding to the photon output, is inversely proportional to the relative amount of estradiol in the sample. A single establishment involves 25 μL of serum, and the stated working range of the test is 20 to 2000 pg/mL. The antibodies employed in the Immulite estradiol assay are secondary to the estradiol molecule at the specificity-enhancing sixth position. The improved specificity of this 17 β-estradiol antiserum makes quantification of estradiol amounts more precise. Reference ranges were used to define women with low estradiol levels (<60 pg/mL) and women with normal concentrations of estradiol (≥60 pg/mL) [26, 27].

### Bone mineral density measurements

Bone mineral density determinations were carrying out at the non-dominant proximal femur (FNBMD), the lumbar spine (L1-L4) (LSBMD), and on the whole body (TBMD) with a dual-energy X-ray absorptiometry (DXA, Lunar DPX NT instrument). The guidelines established by the International Society of Clinical Densitometry were strictly followed [28, 29]. Densitometry technicians performed all BMD measurements using standardized procedures. Customary calibration of the densitometer was accomplish every day by the phantom delivered by the manufacturer; specialists guaranteed that the day-to-day coefficient of variation (CV) was lower than 1.5 %. DXA measurements are presented as grams of hydroxyapatite per square centimeter. For the present study, the sample of females was divided into two categories according to the standards suggested by the World Health Organization (WHO): normal if T-score > − 1.0 and low bone mineral density; defined as follow: osteoporosis if T-score ≤ −2.5; and osteopenia if −2.5 < T-score ≤ −1.0 [30].

### Statistical analysis

We conducted a descriptive analysis of the principal characteristics of interest of the study population. We contrasted predefined risk factors of BMD and levels of BMD between low and normal levels of serum estradiol in premenopausal women. We used the t-student to compare continuous variables with a normal distribution. While, in continuous variables with non-normal distributions, the Mann–Whitney *U* test was used. In addition, to compare categorical variables between groups (low vs. normal) chi square test was computed.

To assess the effects of levels of estradiol and other risk factors on bone mineral density, we computed multiple linear regression analysis.

We computed adjusted odds ratio (OR) and 95 % confidence interval (95 % CI) by means of multiple logistic regression models to determine the size of the relationship between low levels of serum estradiol and other risk factors and bone mineral density.

*P* values lower than 0.05 were thoughtful as statistically significant. All analyses were conducted with STATA software, version 13.0 (Stata Corp LP, College Station, Tex, USA).

## Results

A description of subjects’ characteristics is shown in Table 1. Participants’ mean age was 43.8 years (SD = 2.6), and their mean BMD was 1.18 (SD = 0.08) g/cm^2^. Premenopausal women with low levels of serum estradiol represented 38.9 % of the study population. Statistically significant differences in mean BMD between women with low levels of estradiol and normal levels of estradiol (1.16 vs 1.19 g/cm^2^, *P* = 0.03) were found. The study population’s mean BMI was 26.8 kg/m^2^; 64.5 % of the population had BMI > 25.0, 43.0 % were overweight, and 21.5 % had some grade of obesity. On average, women with normal levels of estradiol had slightly higher BMI than women with low levels of estradiol (26.9 vs. 26.7 kg/m^2^). In addition, women with normal levels of estradiol had slightly higher dietary vitamin D intake than women with low levels of estradiol (211 vs. 235 IU/day); however, this difference was not statistically significant.

**Table 1:** Characteristics of the study population by levels of serum estradiol

	Low estradiol	Normal estradiol	Total	*P-value* ^b^
	(*n* = 104)	(*n* = 166)	(*n* = 270)	
BMD (g/cm^2^) ^a^	1.16 (0.09)	1.19 (0.09)	1.18 (0.09)	0.03
LSBMD (g/cm^2^)	1.18 (0.14)	1.23 (0.15)	1.21 (0.15)	0.005
FNBMD (g/cm^2^)	1.02 (0.12)	1.07 (0.13)	1.05 (0.13)	0.001
Age (years)	44.2 (2.7)	43.5 (2.6)	43.8 (2.6)	0.05
Age at menarche (years)	12.7 (1.5)	12.5 (1.6)	12.6 (1.5)	0.2
Number of pregnancies	3.0 (1.5)	2.6 (1.2)	2.8 (1.3)	0.05
Breastfeeding (months)	9.6 (10.1)	9.3 (10.1)	9.4 (10.4)	0.8
Use of contraceptives (months)	4.7 (14.7)	6.9 (19.8)	6.1 (18.0)	0.3
Days after menstruation	7.5 (1.7)	7.7 (1.8)	7.6 (1.7)	0.9
BMI (k/m^2^)	26.7 (4.2)	26.9 (4.2)	26.8 (4.2)	0.6
Physical Activity (METS/day)	2.4 (2.7)	2.5 (2.1)	2.5 (2.5)	0.7
Tobacco smoking (%)	19.1	16.8	18.2	0.7
Family history of osteoporosis (%)	21.3	17.6	19.3	0.7
Dietary factors				
Energy intake (kcal/day)	2153 (1008)	2072 (836)	2104 (905)	0.4
Dietary calcium (mg/day)	1012 (537)	1162 (560)	1031 (546)	0.4
Dietary vitamin D (IU/day)	211 (133)	235 (154)	226 (146)	0.2
Caffeine (mg/day)	81 (95)	121 (159)	106 (139)	0.02
Alcohol (g/day)	3.1 (6.1)	1.6 (2.8)	2.2 (4.4)	0.01

*BMD (g/cm* ^*2*^ *)* total bone mineral density, *LSBMD (g/cm* ^*2*^ *)* lumbar spine bone mineral density, *FNBMD (g/cm* ^*2*^ *)* femoral neck bone mineral density

^a^ Mean value (SD). Only smoking and family history of osteoporosis are values expressed as percentage

^b^ *t*-test for quantitative variables and chi2-test for qualitative variables

According to the WHO classification of osteopenia and osteoporosis, the prevalence of premenopausal women with osteopenia was 30.1 %, and the prevalence of osteoporosis was 2.1 % (data not shown). The average age at first occurrence of menstruation in our study population was 12.6 years (SD = 1.5 years), and the average number of gestations was 2.8 (SD = 1.3). The study population’s cumulative mean time of breastfeeding was 9.4 months (SD = 10.4 months), and their mean duration of contraceptive use was 6.1 months (SD = 18.0 months).

The effects of age-adjusted level of serum estradiol and other risk factors on BMD are shown in Table 2. In the linear regression analysis levels of estradiol was positively associated with total, lumbar spine and femoral neck BMD (TBMD β = 0.11, *P* = 0.05, LSBMD β = 0.16, *P* = 0.01 and FNBMD β = 0.14, *P* = 0.02). Furthermore, BMI was also positively related to total BMD (β = 0.39, *P* < 0.001), lumbar spine BMD (β = 0.25, *P* = 0.001), and with femoral neck BMD (β = 0.40, *P* < 0.001). Additionally, dietary vitamin D intake (TBMD β = 0.17, *P* = 0.004, FNBMD β = 0.26, *P* = 0.01, and LSBMD β = 0.14, *P* = 0.02), and physical activity (TBMD β = 0.12, *P* = 0.02, and FNBMD β = 0.15, *P* = 0.01), were each positively associated with BMD.

**Table 2:** Effect of levels of serum estradiol and lifestyle factors on BMD in premenopausal women

	TBMD (g/cm^2^)		LSBMD (g/cm^2^)		FNBMD (g/cm^2^)	
	Coefficient β^a^	*P*-value	Coefficient β^a^	*P*-value	Coefficient β^a^	*P*-value
Estradiol (per 10 pg/μL)	0.11	0.05	0.16	0.01	0.14	0.02
Age (years)	−0.08	0.1	−0.12	0.04	−0.15	0.01
Age at menarche (years)	−0.04	0.4	−0.01	0.8	−0.03	0.6
Number of pregnancies	−0.01	0.8	−0.03	0.6	0.04	0.9
Breastfeeding (months)	−0.09	0.3	−0.04	0.5	−0.05	0.4
Use of contraceptives (months)	0.04	0.09	0.03	0.6	0.09	0.1
BMI (k/m^2^)	0.39	<0.001	0.25	<0.001	0.40	<0.001
Physical Activity (METS/week)	0.12	0.02	0.08	0.2	0.15	0.01
Tobacco smoking (number of cigarretes)	−0.14	0.3	−0.02	0.8	−0.29	0.03
Family history of osteoporosis (yes)	−0.02	0.7	−0.02	0.4	−0.01	0.8
Dietary factors						
Energy intake (per 100 kcal/day)	0.10	0.9	0.09	0.9	0.03	0.6
Dietary calcium (per 100 mg/day)	0.06	0.3	0.09	0.1	0.08	0.2
Dietary vitamin D (per 100 IU/day)	0.17	0.004	0.26	0.01	0.14	0.02
Caffeine (per 100 mg/day)	−0.14	0.02	−0.13	0.03	−0.11	0.06
Alcohol (g/day)	−0.06	0.3	−0.03	0.5	−0.07	0.2

*BMD (g/cm* ^*2*^ *)* total bone mineral density, *TBMD (g/cm* ^*2*^ *)* whole body bone mineral density, *LSBMD (g/cm* ^*2*^ *)* lumbar spine bone mineral density, *FNBMD* femoral neck bone mineral density, *BMI* body mass index (normal <25.0 kg/m^2^, overweight ≥ 25.0 - <30.0 kg/m^2^, obesity ≥ 30.0 kg/m^2^)

^a^ Coefficients obtained by multiple linear regression analysis

In the logistic regression analysis, the odds of low BMD increase significantly when the premenopausal females had low levels of serum estradiol (OR = 4.93; 95 % CI: 2.1, 11.4), followed by caffeine intake (OR = 2.6; 95 % CI: 1.2, 6.0), and tobacco use (O =1.97; 95 % CI: 1.05, 3.72). The odds of low bone mineral density decrease according to higher vitamin D intake (OR = 0.98; 95 % CI: 0.97, 0.99). Moreover, women with overweight (≥25.0 - <30.0 kg/m^2^) had lower odds of low bone mineral density (OR = 0.44; 95 % CI: 0.22, 0.91) (Table 3). Further analysis of interaction between smoking status and levels of serum estradiol revealed that the effect of tobacco use increase in the low serum estradiol group (data no shown).

**Table 3:** Odds ratio of low bone mineral density according to levels of serum estradiol and lifestyle factors

	Low bone mineral density^b^		
	OR^a^	(CI 95 %)	*P*-value
Low estradiol (<60 pg/μL)	4.93	(2.14, 11.37)	<0.001
Tobacco smoking (reference: non-smokers)	1.0		
Tobacco smoking (ex-smokers and smokers)	1.97	(1.05, 3.72)	0.01
Age (reference: 40–42 years)	1.0		
43-45 years	1.55	(0.74, 3.27)	0.2
46-48 years	1.95	(0.92, 4.13)	0.08
BMI (reference: < 25.0 kg/m^2^)	1.0		
BMI (≥25.0 a < 30.0 kg/m^2^)	0.44	(0.22, 0.91)	0.03
BMI (>30.0 kg/m^2^)	0.47	(0.20, 1.12)	0.1
Number of pregnancies	1.46	(0.60, 3.53)	0.4
Use of contraceptives (reference: not use)	0.82	(0.42, 1.58)	0.5
Breastfeeding (reference: not bresastfeeding)	1.43	(0.56, 3.65)	0.5
Physical Activity (METS/week)	0.87	(0.74, 1.02)	0.07
Family history of osteoporosis (reference: yes)	1.34	(0.62, 2.86)	0.4
Energy intake (kcal/day)	1.00	(0.9, 1.1)	0.08
Dietary calcium (mg/day)	0.82	(0.40, 1.80)	0.6
Dietary vitamin D (IU/day)	0.98	(0.97, 0.99)	<0.001
Caffeine (>300 mg/day)	2.6	(1.2, 6.0)	0.03
Alcohol (g/day)	0.95	(0.90, 1.02)	0.2

^a^Odds ratios derived by logistic regression analysis. ^b^ Low bone mineral density defined as: (osteoporosis if T-score ≤ −2.5; and osteopenia if −2.5 < T-score ≤ −1.0)

## Discussion

While low bone mineral density (osteopenia/osteoporosis) is considered as a global public health problem, however, its epidemiology in premenopausal women persists unclear. The present study denotes an original contribution to the area of low bone mineral density in premenopausal Mexican females. We found a negative relationship between low levels of serum estradiol and low bone mineral density. Furthermore, we found that not only advancing age, but also tobacco use, and caffeine intake, were determinants of low bone mineral density. On the other hand, vitamin D intake, physical activity, and higher BMI may represent a protective factor, however, BMI has been clearly identified as a cardiovascular and metabolic risk factor.

Our findings are coherent with prior reports, which showed that advancing age was related to small levels of bone mineral density [31, 32]. According our data, each year rise in age was projected to cross-sectionally decline 0.6 % in lumbar spine (LSBMD) or 0.8 % in femoral neck bone mineral density (FNBMD) (data no show). This estimate is consistent with the previous finding that a decrease of 0.8 % in LSBMD and FNBMD occurs annually [32]. In addition, we found that females in the oldest age group (46–48 years) had double the risk of lower bone mineral density compared with the youngest study participants (40–42 years) [OR = 2.; 95 % CI: 0.92, 4.13].

BMI appears to be an additional determinant of bone mineral density, as we found that BMI is positively associated with bone mineral density (β = 0.39; *p* < 0.001). Additionally, we found that overweight females had 56 % less risk of low BMD contrasted with females who had a BMI lower than 25.0 kg/m^2^. This result is consistent with previous findings of a positive relationship between BMI and bone mineral density [33–35]. BMI’s effect on BMD might be determined via blood estrogen levels [36], and may be explained by peripheral aromatization of androgens to estrogens in fat tissue; however, it does not seems to be the mechanism because we adjusted by estrogen levels in the study.

Unlike the cases of weight and age, the impact of tobacco use on bone mineral density has not been conclusively recognized. We tested how tobacco use effects bone mineral density, or the risk of low bone density, in premenopausal women, and found that current or past cigarette consumption was significantly associated with decreased FNBMD. The odds ratio that a current/past smoker would have low bone mineral density was 1.97 (95 % CI: 1.05, 3.72). However, a meta-analysis suggests that cigarette smoking has no detectable effect on bone mineral density [8]. This risk increased in women with low estradiol levels, what makes postmenopausal women a higher susceptibility group.

Equal to this paper previous studies had shown a negative association between caffeine (>2 cups/day or 200–300 mg caffeine/day), and BMD in most of the skeletal. The mechanism proposed is by increasing urinary excretion of calcium and a possibly reduced endogenous Ca absorption. This association deleterious effect of caffeine has been noted when dietary calcium is low [9, 11].

Estradiol has been extensively studied as a prognostic factor of bone health among postmenopausal females, although this relationship has not been thoroughly studied in premenopausal women. We found an important association between serum levels of estradiol and total bone mineral density (β = 0.11; *P* = 0.05). In addition, our data show that subjects with low concentrations of serum estradiol (<60 pg/μL) had 4.93 times greater odds of low bone mineral density compared with subjects who had normal concentrations of serum estradiol (≥60 pg/μL). Estradiol could create positive bone effects through multiple potential pathways; it diminishes initiation of bone metabolic units, it antagonizes parathyroid hormone stimulus of bone reabsorption, it also may improve the survival of osteoblasts via local cytokines or other growth factors, and it enhances the efficiency of gastrointestinal calcium absorption and renal calcium conservation [37]. Some or all of these actions may be responsive to low levels of estradiol. Our data support estradiol’s effect on the reduction of bone mineral density. While estradiol could indirectly influence bone density through body weight, we adjusted our analysis for body weight and still found a strong association.

The results of the present study must be understood with context of some restrictions. First, we could not measure the peak bone mass, bone turnover markers, which represent the optimal bone mass and function of an individual. In this study, the stage of the menstrual cycle, which could influence the assessment of serum estradiol levels for each woman, was not determined. Though, as suggested in a previous study [38], the intra-individual variation was measured in the statistical analysis as an element of the random error in the multiple linear regression models. The association we found between levels of serum estradiol and other lifestyle risk factors and bone mineral density cannot be interpreted as causal, because the study was a cross-sectional investigation. This study was carried out in a group of working class, seemingly healthy female workers in a specific site in Mexico. While these women cannot be considered representative of the Mexican female population as a whole, they may be considered representative of middle to low income female adults residing in the urban areas of central Mexico. Furthermore, we only include specific women of the HWCS, which may introduce a selection bias; however, the women included in this study have similar characteristics compared with the females that were not incorporated in our final analysis.

## Conclusions

In summary our results propose that, rising age, body mass index, tobacco use, caffeine and vitamin D consumption, are significant factors of bone mineral density in premenopausal Mexican women. Additionally, our data suggest that estradiol is a significant determinant of total bone mineral density, lumbar spine and femoral neck in premenopausal Mexican females, which is coherent with the theory that estrogen is an important hormone in the maturation, mineralization and maintenance of the bone in females. More prospective investigations are necessary to clarify the clinical implication of premenopausal women circulating estradiol on subsequent fracture risk. Finally, our results can possibly provide evidence in the direction of the elaboration of more effective public policies for the health encouragement and osteoporosis prevention in Mexican female population.

## Abbreviations

95 % CI: 95 % confidence interval
BMD: Bone mineral density
BMI: Body mass index
CV: Coefficient of variation
DXA: Dual-energy X-ray absorptiometry
FFQ: Food frequency questionnaire
FNBMD: Femoral neck bone mineral density
HWCS: Health Worker Cohort Study
IMSS: Instituto Mexicano del Seguro Social
INSP: Instituto Nacional de Salud Pública
IPAQ: International Physical Activity Questionnaire
ISCD: International Society of Clinical Densitometry
LSBMD: Lumbar spine bone mineral density
METS: Metabolic equivalents
OR: Odds ratio
TBMD: Total bone mineral density
UAEMex: Universidad Autónoma del Estado de México
WHO: World Health Organization
